# Snail meat consumption in Buea-Cameroon: exposures to foodborne pathogens through social practices assessed in 2019 and 2021

**DOI:** 10.1186/s13690-022-01009-8

**Published:** 2022-12-23

**Authors:** Mary Nkongho Tanyitiku, Graeme Nicholas, Jon J. Sullivan, Igor C. Njombissie Petcheu, Stephen L. W. On

**Affiliations:** 1 Department of Wine, Food and Molecular Biosciences, Faculty of Agriculture and Life Sciences, Lincoln University, RFH Building, Room 081, Lincoln, 7647 New Zealand; 2 Department of Pest-Management and Conservation, Lincoln University, Lincoln, New Zealand; 3 Global Mapping and Environmental Monitoring, Yaounde, Cameroon

**Keywords:** Edible land snails, Natural habitats, Foodborne pathogens, Local practices, In-depth settings

## Abstract

**Background:**

Snail meat is an important source of nutrition in Cameroon, but the food safety risks are poorly understood. We characterized public health risks from snail meat consumption as a social system in Cameroon, by examining local snail practices that expose snail meat handlers and consumers to foodborne pathogens.

**Methods:**

We used exploratory qualitative approaches, that is, lived experience, face-to-face in-depth interviews, participant observation and a focus group, to explore fifteen key informants’ routines and lived experiences, and perceptions of two health officials on the food safety practices around snail meat consumption in Cameroon. This information was organized and interpreted using Soft Systems Methodology and Social Practice Theory, which permitted a systemic appreciation of local practices.

**Results:**

We distinguished five kinds of actors (snail vendors, market sellers, street vendors, street eaters and home consumers), who performed seven successive practices (picking, selling, cracking, washing, cooking, hawking and eating). We then identified three worldviews about snails: family support or to reduce poverty, a source of nutrition and a food choice (taste, preference). Our findings revealed participants’ competences were based on childhood learning and ‘inborn’ experiences, and materials used in snail activities reflected participants’ parentage and ‘state of poverty’. Although most interviewees highlighted ‘unhygienic conditions’ when explaining snail picking locations, participants believed washing and cooking should kill all contaminants.

**Conclusion:**

Several opportunities for human exposures to foodborne pathogens including snail picking in domestic wastes and sewage, the selling of unpackaged live snails, improper snail meat washing and hawking in loosely closed buckets, were apparent from our analysis. These findings suggest fruitful opportunities aimed at improving health outcomes among African snail meat handlers and consumers.

## Background

The World Health Organizations’ first global and regional disease estimates in 2015 revealed 420,000 deaths per year from unsafe food [1, 2]. These estimates further showed low-income countries bear the greatest burden, with nearly 70 % of deaths originating from diarrheal disease agents [1, 2]. Due to lack of evidence on the burdens and benefits of tackling food-related illnesses in WHO African regions, food security policies and initiatives have often given scant attention on emerging foodborne diseases [1, 2]. With few processed and packaged foods, large volumes of raw and fresh foods are traded by vendors who possess limited awareness on food safety and hygiene [3]. An example of such foods are African land snails - an affordable source of nutrition and livelihood to many inhabitants in Africa [4–8].

Snail meat is higher in protein (37–51%) when compared to guinea pig (20.3%), poultry (18.3%), fish (18%), cattle (17.5%), sheep (16.4%) and swine (14.5%) [9]. It is low in fats and carbohydrates, contain no cholesterol and are good sources of iron, magnesium, calcium and zinc [7, 9]. With rising awareness on the nutritional benefits of edible snails, there have been an increase in demand by local consumers (that is, in restaurants, hotels, street food industry and food festivals) and foreign exporters (USA, France, Australia, South Korea, among others) [5]. For instance, in Ghana, demand currently outstrips supply [5]. Kaldjob et al. [10] mentioned 76.30% out of 211 individuals consume snail meat in Fako division, Cameroon. Internationally, the African snail species fetches about one third the price of European *Helix* species leading to hundreds of millions of US dollars’ worth of African snail meat exported annually [5, 11].

Despite this significant demand for snail meat, commercial snail farming or heliciculture hardly exists in Africa. Snails are gathered from the environment including forest, farms, gardens, bushes, backyards, footpaths, roadsides, especially during the wet seasons [4–8]. Previous studies have established terrestrial snails as a credible source of infection based on enumerated pathogenic microorganisms [7, 8, 12]. Important pathogens include *Staphylococcus aureus, Salmonella* and *Listeria* species [11].

However, local snail consumption activities which could lead to foodborne illnesses through handlers and consumers’ exposures to potential foodborne pathogens remain under-researched. These local practices from snail gathering, handling to consumption have been largely ignored, rendering the identification of health risk factors, and therefore targeted interventions difficult.

To improve global health among snail meat handlers and consumers, the research reported in this paper explored local practices that could lead, through exposure to snail-borne pathogens, to human illness. An understanding of how human practices underpin exposure to the pathogen hazard provides a basis for developing practical interventions to improve health outcomes, namely focus on relevant social practices.

We consider real-world situations in Buea - Cameroon, and specifically:

1. Describe snail meat consumption in Buea as a system of activities;
2. Identify routine practices in snail consumption activities in Buea;
3. Explore participants’ perceptions on handling and consuming snail meat, and on potential health risks in such snail activities; and
4. Propose opportunities to develop interventions to mitigate these health risks.

## Methods

The approaches used to attain this research objectives are described below in six sections, including, the study area, nature of the study, eligibility of participants, recruiting and engaging participants, theoretical frameworks and interpreting the study findings. The methodological decisions arrived-at and the challenges encountered in carry-out this research are detailed in [13].

### Study location

The study was carried out between June 10th, 2019, and August 24th, 2021, in Buea. Buea is the capital of Fako division, and the Southwest regional headquarter of Cameroon. Buea Municipality is bounded to the north by tropical forest on the slope of mount Cameroon (4100 m above sea level) precisely on latitudes 4°12′N and longitudes 9^0^12′E. The mountain range extends to the sandy beaches of the Atlantic Ocean. The town also shares boundaries with other major towns: the City of Limbe to the Southwest, Tiko municipality to the Southeast, Muyuka municipality to the East and Idenau district to the West [14]. Buea has an estimated population of over 300,000 inhabitants within a highly complex community caught between a blend of urban, semi urban, rural and traditional settings [14]. Bakweri ethnic groups are the majority in the indigenous villages whereas the urban spaces and larger villages are a cosmopolitan blend of more than a hundred local and national inhabitants [14]. Important foreign population especially the Igbos from Nigeria could be found scattered in lucrative commercial activities and farming yams on the rich volcanic soils [14].

Typical in the tropical rainforest and savannah zones of west and central Africa, Buea has an equatorial climate characterized by annual rainfalls of 300-5000 mm, a relative humidity of 70–80% and a temperature range of 20-32 °C [6, 10, 14, 15] It is made up of an evergreen tropical ecosystem ranging from vegetation thick forest, secondary forest, shrubs to savanna towards the peak of the mountain, which has favoured the abundant survival of snails [6, 10, 15]. We were interested in four urban spaces (locations) and road junctions: Buea station, Great Soppo, Molyko/Mile 17 axis, Muea, including participants’ arable land^1^ and homes. These densely populated locations in Buea contained a high availability of key informants,^2^ which we identified as snail collectors, market sellers, roadside street vendors, home consumers and street eaters.

### Nature of the study

We sought to understand foodborne pathogen exposures among snail meat handlers and consumers in the unknown disease-causing situation. Subsequent work [16] clarified the prevalence of pathogens in snails sampled locally. To attain this, we treated key actors as ‘carriers of practices’ [17] and explored routines, lived experiences and opinions of key informants using qualitative research methods. We used face-to-face in-depth interviews [18, 19], participant observation [19], lived experience [18] and a focus group [18, 19] to respectively ensure triangulation^3^ and member checking. Contrary to quantitative research, which seeks to confirm research hypotheses, this exploratory approach provides insights on the ‘human side’ of an issue, social systems and practices, including behaviors, beliefs, opinions and relationships among individuals [18, 19]. The above combination of qualitative methods, with the theoretical framework to be detailed below, was chosen for its potential to understand social systems and practices involved in snail meat as food in Buea, Cameroon, and thus to inform practical interventions to reduce health risks.

### Nature and eligibility of participants

Fifteen women and two health officials (a veterinarian and an epidemiologist^4^) participated in this research. This sample size facilitated the researcher’s close association with each participant which enhances fine-grained, in-depth inquiry in naturalistic settings [20, 21]. Our focus was on snail meat consumption as a social system rather than on the act of consumption itself. We sought to understand the social systems and practices that could result in human exposure to snail-borne pathogens. Exposure to such pathogens at the time of consumption would be a product of practices involving collection, handling and preparing the snail meat. Because of that focus, our informants to understand local practices were women. The focus on women was based, in part, on findings which revealed women as the backbone in Cameroons’ snail businesses [15, 22]. Ngenwi et al. [22] mentioned 60% of rural women took part in snail activities in west and central Africa, and 42–62% of their rural income was derived from the sale of snails. We selected women who possess childhood experiences in handling snails, and who still incorporate snails in their routines.iteria for participation required participants that have lived in Buea for at least five years and were actively engaged in snail collection or consumption activities. Furthermore, we focused on women that were also housewives and responsible for preparing food for their families. In most cases, those who collected snails for selling or hawking^5^ were also consumers; only three of the 17 participants stated they did not collect snails personally and only obtained their snails as ready-to-eat product. The researcher used local knowledge through lived experience (since she had grown up in the setting), and preliminary field observations to ensure the participants as a whole covered each stage in snail meat consumption.

### Recruiting and engaging participants

We were guided by three interview instruments: an interview protocol [18], an information sheet^6^ and a written consent form. These instruments were designed to manage confidentiality, participants’ vulnerability (including living in a setting of poverty, fear and suspicions resulting from the Anglophone crisis^7^), and any misconceptions of this research purpose. To ensure confidentiality, participants were repeatedly reminded not to reveal their identity during the interviews, and as no two interviews were held on the same day, interviews were identified by its date. With regards to vulnerability, when we came across a key informant in a market, we observed at approximately fifty meters, then approached them politely and introduced our research purpose. In some cases, we initiated conversations while buying snails from key informants. In parallel with participant observation, note taking and recruiting participants, the researcher and note taker^8^ started by visiting local markets, road junctions and streets. We primarily used the Cameroon Pidgin English (CPE)^9^ and participants needed to possess a working telephone to contact us during a one-year withdrawal period. Prior to interview dates, participants were handed copies of an information sheet and consent form, to enable them to seek advice or further clarification at their discretion.

Face-to-face interviews of twenty-five to sixty minutes per participant were conducted at participants’ homes and convenience in the presence of trusted relatives and friends. In line with local customs, it was unethical to tell participants relatives to leave as we were strangers at their homes. In as much as relatives clarified doubts to participants, they created useful discussions that were integrated in our findings. It should be noted that trust is gained when a researcher is honest about her intentions and expectations, considers the cultural settings and highlights the benefits of the study to participants [23].

### Theoretical frameworks

To understand health risks in a social setting, Buea, and in an undefined food-disease situation as edible snails, we chose two theoretical frameworks: Soft Systems Methodology [24] and Social Practice Theory [25].

Soft Systems Methodology (SSM) was chosen for its capacity to structure and conceptualize complex human situations. Contrary to hard systems with well-defined problems, soft systems strive to learn from the complexity of a ‘mess’ through different perceptions and worldviews that exist in the minds of different people involved in a situation [24, 26]. Further, we use SSM within the tradition of critical systems thinking [17], meaning we are not claiming that our observations can be accounted for by a systems analysis, only that viewing practices as if they are expressions of multiple interactions, perspectives and judgements is a sound way to explore complex social practices [17, 27]. SSM in this research was used as a descriptive tool to gain an appreciation of interacting influences, and differing perceptions, judgments and values. As outlined by Proches and Bodhanya [26], we used rich pictures, root definitions, CATWOE and conceptual models as a four-staged SSM approach. Each of these elements is now elaborated. A rich picture, in SSM, is way of representing the complexity of human situations with multiple interacting relationships. Usually, in SSM, it is done as a literal picture. In our case we chose to visualize the social complexity of the situation through an Ishikawa diagram. That choice is discussed further under our results and in [13]. Root definitions (RD), in SSM, are a standardized systemic description of a system. An RD describes succinctly an action, how it is performed and its intended purpose. In our case RDs enabled us to describe systems from differing perspectives. CATWOE, is a SSM acronym used for systemic description of purposeful activities. It identifies **c**ustomers (those affected by a transformed situation), **a**ctors (those bringing about a transformed situation), the **t**ransformation (the envisaged difference that would be made), **w**orldview (assumptions that make the transformation make sense), **o**wners (those with control), and operating **e**nvironment (givens that may affect the transformation). This enabled us to recognise and describe the effective systems relating to differing worldviews and desired transformations. Conceptual models, in SSM, are used to represent a purposeful activity system through a set of logical interactions implied by the description of the system, or root definition. They make dependencies discussable. In our case, such a model made clear the range of interlocking activities that constitute the social system underlying snail meat consumption.

Social Practice Theory (SPT) was chosen for its capacity to uncover the routines of snail handlers and consumers. Social practices refer to everyday practices and the way they are typically and habitually performed in a society [25, 28]. It views practices as constellations of elements such as materials, knowledge, skills, emotions, meaning to people as part of their everyday life practices [25, 28–30]. In this study, we used Shove et al. [25] simplified elements of competence, materials and meaning. We considered informants as ‘carriers of practices’ [28], where this implies “they are neither autonomous nor the judgmental dopes who conform to norms: They understand the world and themselves, and use know-how and motivational knowledge, according to the particular practice” [28]. SPT has been used in understanding public health [29, 31] as well as in domestic food safety practices [31]. Meah [31] argued that behavior-based approaches in tackling food safety are simple and limited, as they focus on the ‘what’ and little on the ‘how’ and ‘why’ which are all implicated in what transpires in a kitchen. Given routines contribute to how people handle food, Meah [31], further suggest that it is appropriate to apply a theoretical framework (for example, SPT), to reflect those routines and the embedded nature of what takes place in a domestic kitchen.

**Fig. 1:**
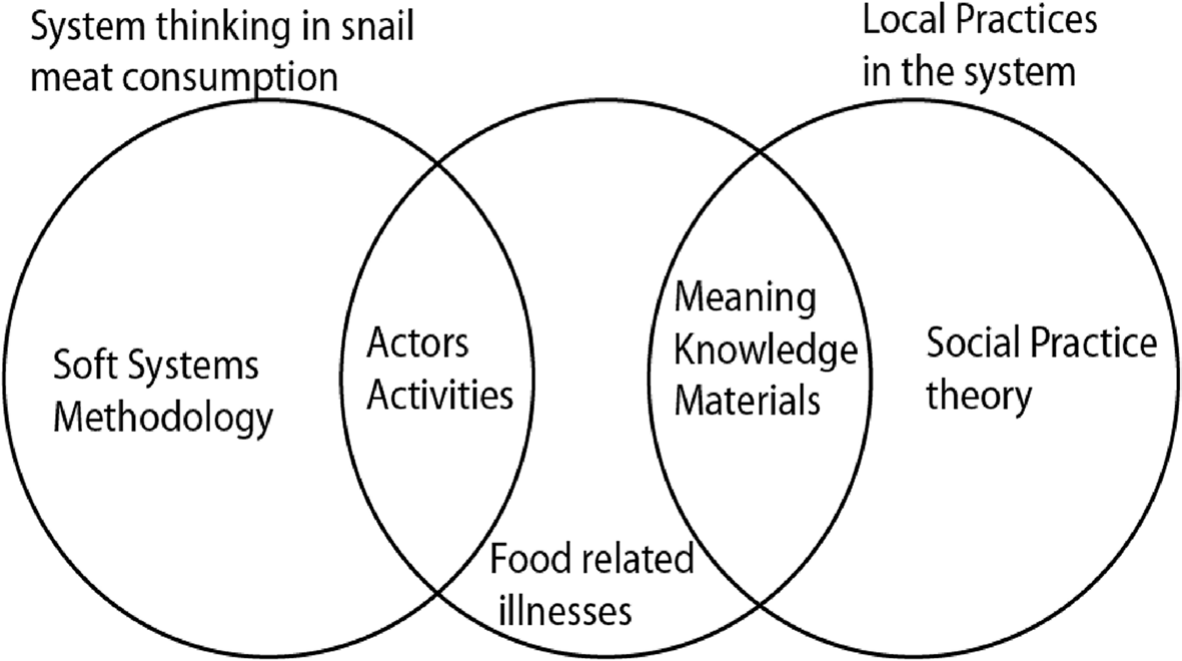
Theoretical framework integrating Soft Systems Methodology and Social Practice Theory

Our integration of SSM and SPT is illustrated in Fig. 1. SSM provided a way to gain and use a ‘systemic’ understanding of the risks. Rather than simply identifying risk factors SSM showed how factors and actions relate to one another, or influence one another. SPT provided a generic framework for interrogating particular practices. It was used to describe practices considered important for understanding human exposure to pathogen hazards associated with consumption of snail meat. We see the two frameworks as very complementary. Each assumes systemic interaction between multiple elements to result in outcomes. Their distinction can be seen in the unit in focus. For SSM it is the experienced mess [24], for SPT some identifiable practice or activity is envisaged. In common between the two approaches is an appreciation of human motivation and action being, in part, products of how actors and communities make sense of their situation; for SSM that is attention to worldview, for SPT it is attention to meaning.

### Interpreting the study findings

Field notes, photos and audio recordings from interviews were treated as information (defined as processed data with specific meaning or ideas). In other words, some degree of interpretation was inherent in the raw material used in reaching our findings. Manual transcription of interviews by the researcher was chosen due to the use of CPE, and inaudible pronunciations registered in the recordings. Through repeated listening and thorough reading of transcripts, we did hand coding and gained deep understanding of the obtained information. Using SSM and SPT, we then made socially robust sense of the information to reach our understanding of human practices that may constitute exposures to foodborne pathogens in snail meat consumption.

## Results

To understand health risks among snail meat handlers and consumers in Buea, we needed to pinpoint: 1) a systemic model of key activities, 2) local practices in snail meat consumption as a system, and 3) participants’ perceptions on snails as food and on potential health risks.

### Systemic model of key activities

To produce a systemic model of key activities, we followed SSM in picturing the problem as a complex, ‘messy’ situation with many contributing and interacting factors, and then identified some specific human activities to model.

### Context and complexity

Information obtained from participant experiences, in-depth interviews, observations and grey literature was developed into a rich picture using an Ishikawa diagram (Fig. 1). Ishikawa diagrams are an approach which thoroughly analyzes situations to identify the possible causes of a problem [32]. This approach permitted us to illustrate the complex potential contributors of health risks in snail consumption activities by demonstrating the possible role of eight kinds of inputs into potential health outcomes from human exposures. With this in mind, we then focused on ‘high salience’ human activities in order to better understand these health risks.

### Identifying and modeling human activities

**Fig. 2:**
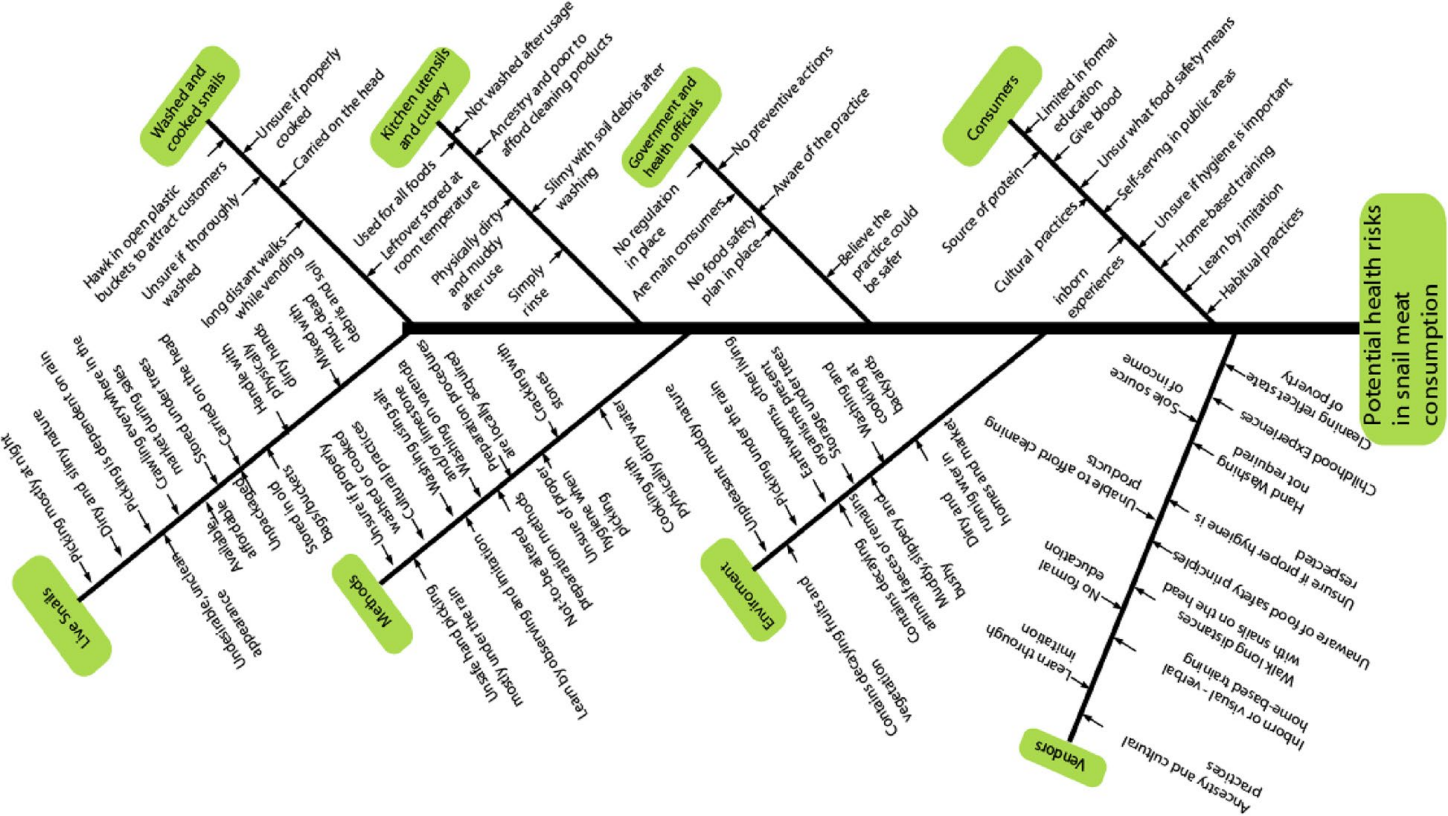
Ishikawa diagram identifying and describing potential health risks to snail meat consumers along the production system

**Fig. 3:**
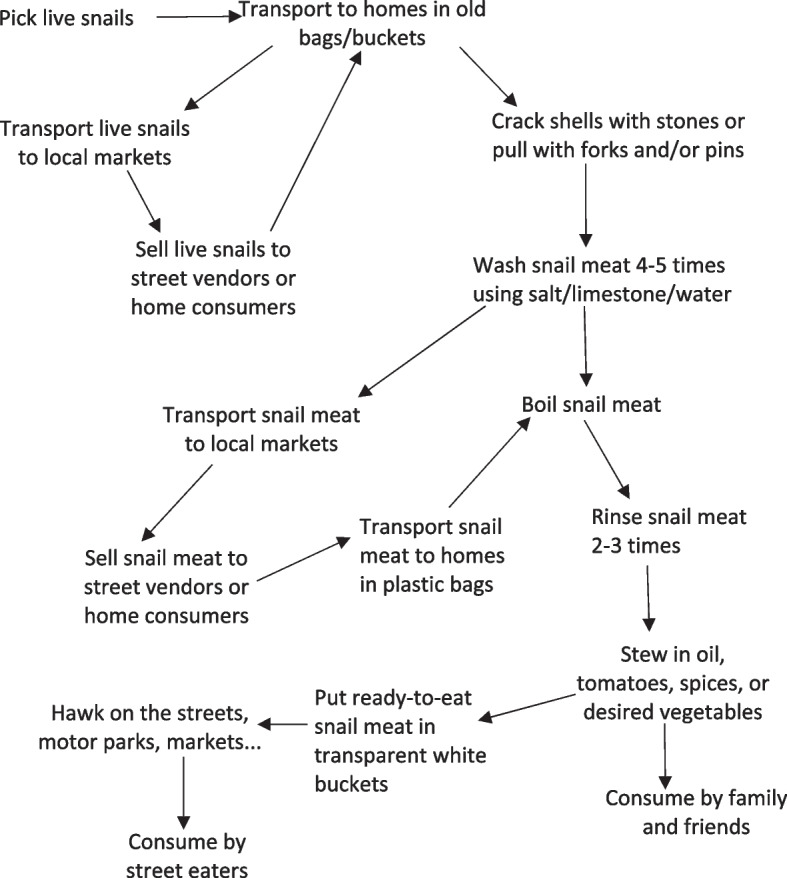
A model of snail consumption activities

To map key human activities in snail meat consumption, we developed a systemic model in Fig. 2, and then in Fig. 3, highlighted the connections (that is, common words in addressing each snail activity), that existed among participant excerpts. For example,

“We **pick** and **wash** every Saturdays and **sell** on Sundays in the market. I **wash** snails every weekend…” (Ms)

“…**picking**, **washing** and **selling** snails have safe my family from many situations […] from hunger” (Sv)

**Fig. 4:**
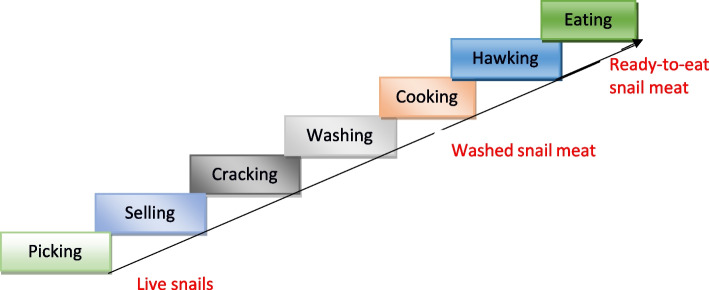
Sequential local practices from live snails to ready-to-eat snail meat

From these common ways of speaking, we distinguished seven interrelated local practices performed in a succeeding order (Fig. 4). For instance, picking, cracking and washing occurred before cooking while hawking and/or eating thereafter.

To reveal different snail activities in terms of our diverse research participants (actors), their purpose of acting or performing each local practice, we used root definitions (Table 1). We came up with five statements using the PQR formula, that is, do P-what, by Q-how, in order to achieve R-why. We then used SSM mnemonic CATWOE to elaborate on these actors and activities (Table 2). Snail consumption activities are defined by a transformation process (T) and worldviews (W) which required actors, women (A) to do the activities which make up T. It will affect these women and street eaters (C) who are beneficiaries or victims (customers of the system). These activities embrace constraints from its environment (E) and the whole transformation can be seen as ‘owned’ (O) by these women, consumers or health officials in that they have the power to change or stop the system.

**Table 1:** Actors in snail consumption activities

Actors		Root definition
1	Snail collectors (Sc)	Collectors who earn money by picking live snails in order to support family or reduce poverty
2	Market sellers (Ms)	Sellers who earn money by selling live snails or washed snail meat in order to support family or reduce poverty
3	Street vendors (Sv)	Hawkers who earn money by hawking ready-to-eat snail meat in order to support family or reduce poverty
4	Home consumers (Hc)	Households who increase nutrient intake by eating snail meat in order to reduce malnutrition
5	Street eaters (Se)	Road users (traders, students, travelers….) who satisfy tastes by eating ready-to-eat snail meat in order to respect regular eating behaviors

**Table 2:** CATWOE of the model system

CATWOE	Description		
	Support family or reduce poverty	Source of nutrition	Tastes, preference
**Customer**	Sc, Ms., Sv	Hc, Ms	Sv, Se
**Actor**	Sc, Ms., Sv	Hc	Se
**Transformation**	Sc, Ms. pick and sell live snails, Sv sell ready-to-eat snail meat to earn money	Hc pick or buy live snails, cook and eat snail meat to increase protein and calcium intake	Se buy and eat ready-to-eat snail meat to satisfy tastes
**Worldview**	Readily available, little or no investment, no skills required	Source of calcium and blood, cheap, affordable, cultural dish	Delicious, satisfying, tasteful, affordable
**Owner**	Sc, Ms., Sv, Hc, Se, health officials	Ms Hc, Health officials	Sv, Se, Health officials
**Environment**	Weather and poverty-driven, increase family needs	Diet-conscious, cultural upbringing, poverty, beliefs	Habits, lifestyle, irresistible tastes

### Understanding local practices and perceptions

Having identified and modelled human activities that make up the system of snails as food in Buea, we now describe the competencies, materiality and meaning-making that influence local practices (and therefore influence the potential for exposure to foodborne pathogens). For this we used SPT. Table 3 presents evidence from participants in Buea on social practice around snails as food and associated hygiene.

**Table 3:** Constraints and drivers that influence snail local practices

Element		Snails as food	Hygiene
Competence		*What competence influences practice? How do people get this knowledge?*	*What do participants know about hygiene when handling and eating snails? How do people learn?*
	Participants’ evidence	Hc: “We have been eating since we were small, so it is but normal, we just know that you want to pick you just go there” [pointing at the back of her house] Hc: It is but obvious, we are growing in a society where we eat snails, we have been eating since we were small, [..], so it is not like there is a particular skill that we have been taught, no, we just know [..]” Sv: “I think for those who have never seen how they are washing it anywhere, they don’t need any expert [..] but if you are somebody who have seen where they are washing, just follow what the people do, [..] no you don’t need any training Researcher: ‘*If you are teaching your daughter to do what you do, what would you teach her?*’ Sc: “I will ask her to go out, look for cold or hidden places, under the grasses where there is no or less sunlight, she will find snails”	Hc: “We don’t really pay much attention to hygienic conditions, besides when you go and pick in very rotten things, [..] people [snail collectors] go to the bush, excrete there and keep it for snails to come and they will pick in the evening, it is funny, but it is the truth” Sc: “To protect yourself from food poisoning, snails need to be washed thoroughly, [..] some people hurry [..] take your time and make something good, for yourself and family” Hc: “For many years, since they gave birth to me, my mother showed me how to wash and how to cook snails to eat in the house” Hc: “You can learn from anyone who does it. I was not trained in a school. She just needs to watch from beginning to end”
	Summary finding	Competence is ‘inborn’, ‘from childhood’, that is, ‘*the way of seeing things being done and imitate’*. It is locally acquired through family and community.	Hygiene is each household ‘state of cleanliness’. It is based on ‘*this is the way I was taught*’. For instance, washing and cooking involve inherited visual and verbal ‘instructions’ initiated from one generation to the next. Preventing diseases or staying healthy depends on washing and cooking practices
Materials		*How do materials influence the picking, handling, and consumption of snails?*	*What technologies and methods influence how people practice hygiene when handling or consuming snails?*
	Participants’ evidence	Sc: “we use very good and shining torches to pick snails; places are very dark, if you do not use a torch that the light is good, you will not see snails” Sv: “…I think it is a nasty method, imagine taking this grinding stone, [..] the one we use to grind pepper, you make crack, those shape particles [snail shells] are piercing your hand and the meat” Researcher: *do you know the time and temperature you cook your snail meat?* Hc: ‘I do not have a thermometer, when I see it ready, I will know by looking at it, [..] you do not need to look at the time as well, a change in colour is enough’	Hc: “[..], in moist areas, around the pit toilet, I will send her [her daughter] there to go and pick it [snails]” Hc: “It [snails] is too slimy, so you must wash it outside because you have to throw the slime” Two street vendors mentioned they will prepare on three-stone fires because ‘*firewood is cheaper than gas’*, and some home consumers preferred firewood due to cultural upbringing
	Summary finding	Materials reflect participants’ ‘inborn’ experiences, parentage and ‘*state of poverty*’. For example, picking at night with torches, transporting live snails in old bags and buckets, pulling snail meat with stainless-steel table forks or ‘*pins’* or cracking snail shells with stones, cooking by visual observation.	Handpicking involves poor or uncontrolled hygiene. Live snails are physically dirty, ‘unhygienic’ containing soil debris, food wastes. Local practices are constrained by the very simple technologies available. For instance, selling live snails in old bags/buckets, washing in two silvery or plastic basins on the veranda, cooking on three-stone fires in front or behind the house and hawking in loosely closed plastic buckets.
Meaning		*What meaning influences people to handle and eat snails?*	*What do people believe about hygiene in relation to snails?*
	Participants’ evidence	Hc: “…. yes, it is really delicious when you prepare it and if you know how to prepare it, so it is something that we love eating, so we like to pick and cook it” Se: “snails are nutritive[..]contains calcium, give us blood”	Hc: “I have never thought of that [hygiene] because even the ones that we buy from the market, hmm at times we do not even know where they pick them, so when you pick there [pointing at a pit latrine], it is not actually the excreta but the meat you are picking, so it is good” Sc: “You must pay attention to the sticky liquid, [..] you need courage to touch snails as it irritates many people” Epidemiologist: “…. once you expose yourself to a dirty environment, like picking from unhygienic conditions means they [Sc, Hc, Ms] are already exposing themselves to diseases”
	Summary finding	Snails support family, it is a source of nutrition and a food choice. Two vendors mentioned: ‘*we need money*’, ‘*it is a moneymaking activity*’.	Although eating snail meat is delightful, snail picking to cooking are ‘unpleasant’ and ‘nasty’ activities. For instance, most participants expressed ‘hmm’ when snail picking and washing were mentioned

In terms of competencies: competence is ‘inborn’, ‘from childhood’, that is, ‘the way of seeing things being done and imitate’. It is locally acquired through family and community. Hygiene is seen as each household’s ‘state of cleanliness’. It is based on ‘this is the way I was taught’. For instance, washing and cooking involve inherited visual and verbal ‘instructions’ initiated from one generation to the next. Preventing diseases or staying healthy is understood to depend on washing and cooking practices.

In terms of materiality: materials reflect participants’ ‘inborn’ experiences, parentage and ‘state of poverty’; for example, picking at night with torches, transporting live snails in old bags and buckets, pulling snail meat with stainless-steel table forks or ‘pins’ or cracking snail shells with stones, cooking by visual observation. Handpicking involves poor or uncontrolled hygiene. Live snails are physically dirty, ‘unhygienic’ containing soil debris, food wastes. Local practices are constrained by the very simple technologies available. For instance, selling live snails in old bags/buckets, washing in two silvery or plastic basins on the veranda, cooking on three-stone fires in front or behind the house and hawking in loosely closed plastic buckets.

In terms of meaning: snails support family economies, as a source of nutrition, a source of income and a food choice. Although eating snail meat is seen as delightful, snail picking to cooking are ‘unpleasant’ and ‘nasty’ activities.

## Discussion

Our purpose was to understand health risks associated with snail-meat social systems and practices in rural settings, particularly in Buea, to inform interventions for improved health outcomes. To our knowledge, we report on yet-to-be documented snail meat disease-causing real-world situations. We fill-in the knowledge gap existing between 1) already enumerated foodborne pathogens associated with snails in farms as well as those sold in local markets [7, 8, 12] and 2) the ways in which local people are routinely exposed to snail-borne pathogens.

Few studies have used qualitative approaches to raise food safety concerns in snail handling processes. For instance, Nyoagbe et al. [7] in a survey mentioned snails sold in Ghana were unpackaged, in an unhygienic state, and slime, shelling and dirt were snail preparation problems. Temelli̇ et al. [11] identified personnel hands and equipment used as secondary snail meat contamination sources during large-scaled frozen snail meat processing. Similarly, Novakovj and Grujj [33] highlighted the importance of hand hygiene, hand washing and disinfection in snail meat processing. We distinguish our findings from the above research as it sought to understand human exposures to food safety hazards by seeing through the eyes of key informants and provide foodborne disease-related evidence of snail-meat practices in traditional settings. Our use of SSM provided a rationale for where to look in a complex social system for risky activities. SPT provided a powerful lens for looking more closely at practices that seem important points of exposure to pathogen hazards (as identified by SSM). Our use of the two frameworks provides a strong lead for the design of future interventions: SSM offers a rationale for which activities to target to improve health outcomes, while SPT offers a more fine-grained rationale for how to intervene for change in chosen activities.

Our findings showed that snails are seen as financial support for families, a source of food to low-income inhabitants, and are preferred to other foods in terms of taste. To reduce protein malnutrition, the African giant land snail has been recommended as a good substitute among the vulnerable group including children, pregnant women, aged and adolescents in Nigeria [4, 9] as well as in our research settings. In the quest to solve undernourishment and protein deficiency challenges, our findings revealed snail meat handlers and consumers become undoubtedly exposed to foodborne pathogens, which could lead to adverse food-related illnesses, especially to the above-mentioned vulnerable group. Competences in carrying out snail activities are locally acquired through family and community and hygiene is based on ‘*this is the way I was taught*’. Snail collectors picked snails and sell to market sellers who then sell to street vendors or home consumers. While home consumers prepared the meat and eat at home with family, street vendors cook and sell ready-to-eat snail meat to street eaters in public areas. We identified potential human exposures to foodborne pathogens, for instance, handpicking in unhygienic habitats (domestic wastes, decaying vegetation, arable land, footpaths, backyards…), the materials used in picking, washing, cooking and consumption (that is, old bags, open buckets, lack of constructed kitchens - cooking in front or at the back of the house on three stone fires, physically dirty and improperly washed kitchen utensils and cutlery…), street hawking in loosely closed buckets and self-serving on roadsides, road junctions, markets and motor parks.

It should be noted that snails and microorganisms are closely associated with habitats characterized as ‘filth’, ‘sewage’ and ‘rotten materials’ [7, 8]. In our study area of Buea, the current waste management has been described as ‘poor’ ‘pathetic’ and ‘rudimentary orchestrated’ which could increase disease prevalence [34]. As such, local practices in snail picking to consumption as well as its improper handling could lead to potential public health risks particularly in Buea and other sub-Saharan countries with comparable snail handling and consumption practices.

Additionally, based on our findings, we initiated a start-up point for further research in an undefined and under-research health risks problem situation using an innovative combination of in-depth interviews, participant observation, lived experience and a focus group. We have conducted a study to determine the prevalence of key enteric pathogens in snails collected from natural habitats, which identifies substantive public health risks to local communities consuming snail meat [16]. Given our findings in this paper about social systems and practices that expose people to snail-borne pathogens, we recommend a) improved systems of snail farming or heliciculture among the local people in sub-Saharan Africa, b) prior to consumption, a starvation (purging) of live snails, which could effectively eliminate ingested excrement and contaminants in snails, and finally c) an initiation of governmental food safety regulation and interventions that target the local people’s cultural snail meat practices. In order to reduce health risks from foodborne pathogens, changes will need to engage with deeply embedded locally acquired competences and worldviews and take account of the material circumstances that configure key local snail-meat practices.

## Conclusion

Locally called ‘Nyamangoro’, ‘Congo meat’ or ‘slow boys’ in Cameroon, African land snails are popularly consumed and traded in west and central Africa. It is a source of food and livelihood to rural inhabitant in the tropical rainforest. With underdeveloped snail farming practices, African giant land snails are typically gathered from natural habitats including forest, farms, footpaths and decaying vegetation. Situating our study in sub-Saharan Africa and particularly in Buea - Cameroon, our purpose was to understand potential health risks in local snail meat picking, handling and consumption activities. This is important given the high incidence of foodborne pathogens we detected in snails sourced from this region [16]. We revealed snail meat handlers and consumers are substantially exposed to foodborne pathogens, thus indicating local snail consumption practices as significant public health risks. Snail picking occurred in locations with poor or uncontrolled hygiene. Snail meat washing, cooking and selling took place in uncertain and possible food-contaminated cultural settings that reflected participants’ parentage and ‘state of poverty’. This research suggests fruitful opportunities for designing and testing interventions aimed at improving health outcomes for families and communities involve in the handling and consumption of edible terrestrial snails.

## Data Availability

The data used and/or analysed during the current study are available from the first author (email: mary_tanyi@yahoo.com) on reasonable request.

## Footnotes

1 Usually, small areas of land used for vegetable cultivation destined for household consumption or for income earning when production is in excess of household needs

2 Key informants are perceived to provide more knowledge about a topic by the nature of their position in a culture, and their information-rich connections [16]

3 Triangulation, member checking ensures credibility, an important element used to establish trustworthiness [20]

4 In Cameroon, epidemiologists assess disease prevalence while veterinarians perform regulatory inspections on food animals.

5 Selling indicates the point of sale is static, most commonly at a roadside location, whereas hawking identifies the point of sale as mobile, whereby goods are transported in a basket on the seller’s head around the locale.

6 An outline of this research purpose and ethical considerations.

7 A marginalization crisis initiated by lawyers and teachers which later lead to armed conflicts with separatist fighters calling for secession of English Cameroon (that is, 20 % of the total Cameroonian population).

8 A female geographer with deep knowledge in qualitative enquiries as well as the landscape of Buea.

9 CPE is a common language spoken in public (markets, streets) as well as informal settings in most countries in west and central Africa especially Cameroon, Ghana and Nigeria.
